# Rangeland Condition Monitoring: A New Approach Using Cross-Fence Comparisons of Remotely Sensed Vegetation

**DOI:** 10.1371/journal.pone.0142742

**Published:** 2015-11-13

**Authors:** Adam D. Kilpatrick, Megan M. Lewis, Bertram Ostendorf

**Affiliations:** School of Biological Sciences, The University of Adelaide, Adelaide, Australia; University of New England, AUSTRALIA

## Abstract

A need exists in arid rangelands for effective monitoring of the impacts of grazing management on vegetation cover. Monitoring methods which utilize remotely-sensed imagery may have comprehensive spatial and temporal sampling, but do not necessarily control for spatial variation of natural variables, such as landsystem, vegetation type, soil type and rainfall. We use the inverse of the red band from Landsat TM satellite imagery to determine levels of vegetation cover in a 22,672km^2^ area of arid rangeland in central South Australia. We interpret this wealth of data using a cross-fence comparison methodology, allowing us to rank paddocks (fields) in the study region according to effectiveness of grazing management. The cross-fence comparison methodology generates and solves simultaneous equations of the relationship between each paddock and all other paddocks, derived from pairs of cross-fence sample points. We compare this ranking from two image dates separated by six years, during which management changes are known to have taken place. Changes in paddock rank resulting from the cross-fence comparison method show strong correspondence to those predicted by grazing management in this region, with a significant difference between the two common management types; a change from full stocking rate to light 20% stocking regime (Major Stocking Reduction) and maintenance of full 100% stocking regime (Full Stocking Maintained) (P = 0.00000132). While no paddocks had a known increase in stocking rate during the study period, many had a reduction or complete removal in stock numbers, and many also experienced removals of pest species, such as rabbits, and other ecosystem restoration activities. These paddocks generally showed an improvement in rank compared to paddocks where the stocking regime remained relatively unchanged. For the first time, this method allows us to rank non-adjacent paddocks in a rangeland region relative to each other, while controlling for natural spatio-temporal variables such as rainfall, soil type, and vegetation community distributions, due to the nature of the cross-fence experimental design, and the spatially comprehensive data available in satellite imagery. This method provides a potential tool to aid land managers in decision making processes, particularly with regard to stocking rates.

## Introduction

Rangelands are areas of managed grazing and are the largest landuse by area, worldwide. Benefits of sustainably managing global rangelands may include a contribution to carbon sinks [1] and reduction in economic impacts of dust storms [2], but defining policies on stock management is difficult due to spatiotemporal variability and the broad scales of these lands. While managers of rangelands cannot control natural variation in landscapes and climate, they can respond to it, through decisions they make in terms of stock management. In order to make sustainable management decisions, land managers need access to information which identifies the effects of their stocking management, while accounting for natural variation in the environment.

Current field-based methods for monitoring rangelands, although widely used, are insufficient because of their expense and the time required to adequately and repeatedly sample over extensive areas [3]. Remotely-sensed monitoring has obvious advantages due to its consistency, comprehensive coverage and broad scale [4]. While vegetation cover levels can be readily and rapidly determined using satellite imagery [5], [6], [7], [8], [9], [10], and [11] it is difficult to use this information to make objective comparisons of management units such as paddocks or fields. Many factors besides management can influence the amount of vegetation cover at any location in space and time, including soil type, landform, vegetation community, and weather patterns such as rainfall. Controlling or even modelling the effects of these variables across rangelands is difficult [12], [13]. While it is possible to compare the condition of paddocks in rangelands using remotely-sensed imagery [3], [14] most methods have limitations because they cannot separate the effect of natural factors causing variation in condition from management influences. The limitations include lack of spatially comprehensive rainfall data or control for other factors that contribute to vegetation condition, such as spatial and temporal variability of rainfall, and landscape variables such as soil type and vegetation communities [15], [12], [16], [17]. One method recently developed to isolate the effects of rangeland management from remotely sensed vegetation cover measures, is the Dynamic Reference-Cover Method [18]. This method identifies reference pixels of persistent high vegetation from a time series of imagery and a regional moving window in order to classify all pixels within the moving window, based on their potential difference in vegetation cover [18]. A second method involves modelling vegetation growth using available rainfall data, which is then subtracted from measured vegetation growth in time series imagery, inferring grazing effects [14], [19], [20].

An alternative method for assessing rangeland management using remotely-sensed data is to use cross-fence sample comparisons [16], building on an approach that has long been used by ecologists and rangeland scientists [21], [22], [23], [24], [25], [26]. This methodology controls for localized variation in environmental factors such as landsystems, vegetation community type, soil type and rainfall. It does this by directly comparing only samples that are immediately adjacent to one another and separated by an identifiable management boundary, such as a fenceline. From there, simultaneous equations are generated representing the condition of each paddock, based on all the cross-fence pairs it shares with its neighbours, and the values of its neighbours. This leaves only historical, past and present grazing management of the paddock and differential grazing distribution attributable to such factors as the piosphere effect [27] and wind direction [28], as the variables responsible for vegetation differences across fencelines. Management of the paddock involves a number of factors, but especially management of stocking intensity, the distribution and availability of waterpoints, and breed of stock selected. These management effects all contribute to the relative ranking of each paddock [16].

This paper presents the first application and validation of the cross-fence comparison methodology in a study area including several pastoral leases in arid South Australia, using remotely-sensed vegetation cover and mapped fencelines between paddocks. Under realistic conditions we evaluate whether the cross-fence methodology shows the differences between paddocks expected from broad management regimes, and whether this method can be used to detect changes resulting from management over time.

## Methods

### Study area

**Fig 1:**
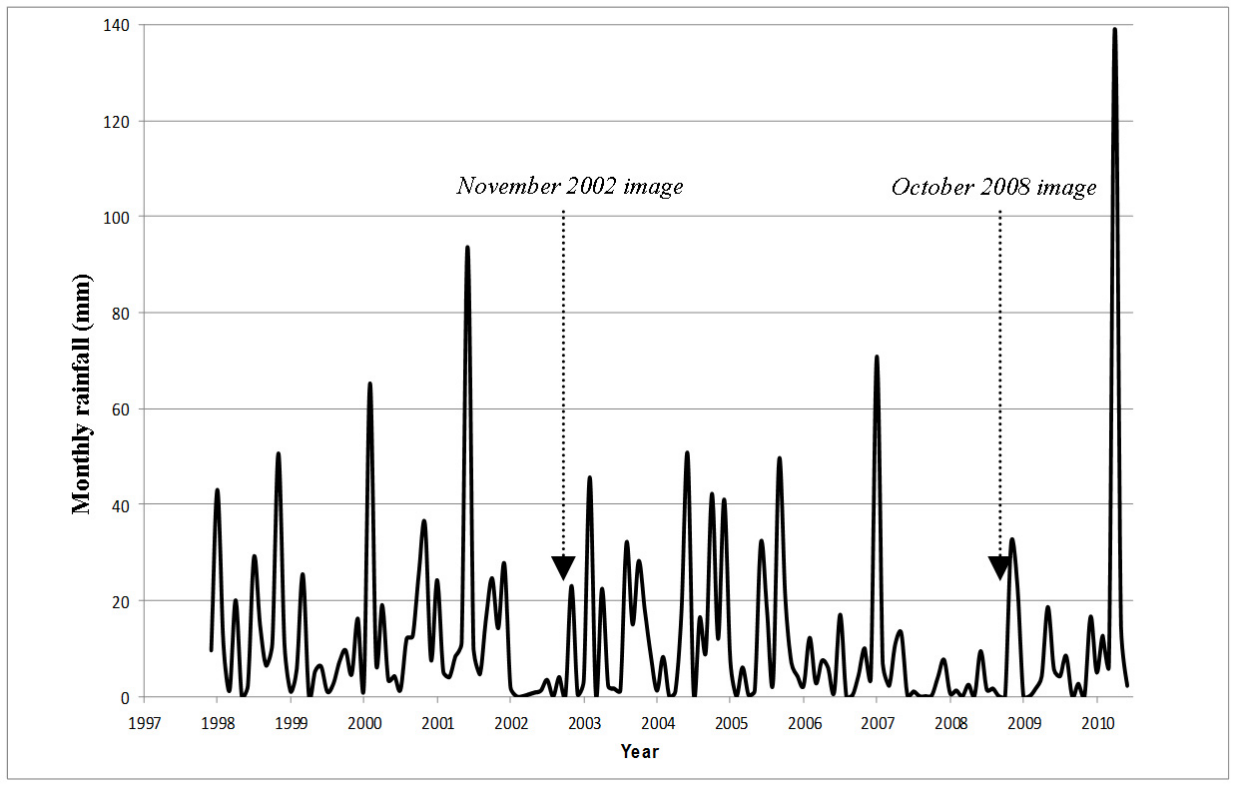
Monthly rainfall at the Olympic Dam Aerodrome (centre of study area), and dates of Landsat TM image acquisition.

The study area consisted of 22,672km^2^ of arid rangeland, in the region south of Lake Eyre and west of Lake Torrens, South Australia. The region contains various land types, including vegetated sand dunes, calcareous chenopod swales, stony plains, cane grass swamps, Mulga (*Acacia aneura* ssp. *aneura*) dominated sandplains, claypans and some breakaway or tableland country. Mound springs of the Great Artesian Basin occur in the northern-most paddocks of the study area. Each landsystem has different characteristic vegetation types and cover levels, and responds differently to grazing pressure. Climate is arid, with annual rainfall averaging 160mm in the south and centre (Fig 1) to approximately 100mm in the north. Annual rainfall is highly variable, both spatially and temporally. The most effective rainfall events are generally in summer, and after droughts which often extend for several years (Fig 1). Summers are generally very hot, whilst winters are cool to mild.

### Study area management history and characteristics

**Fig 2:**
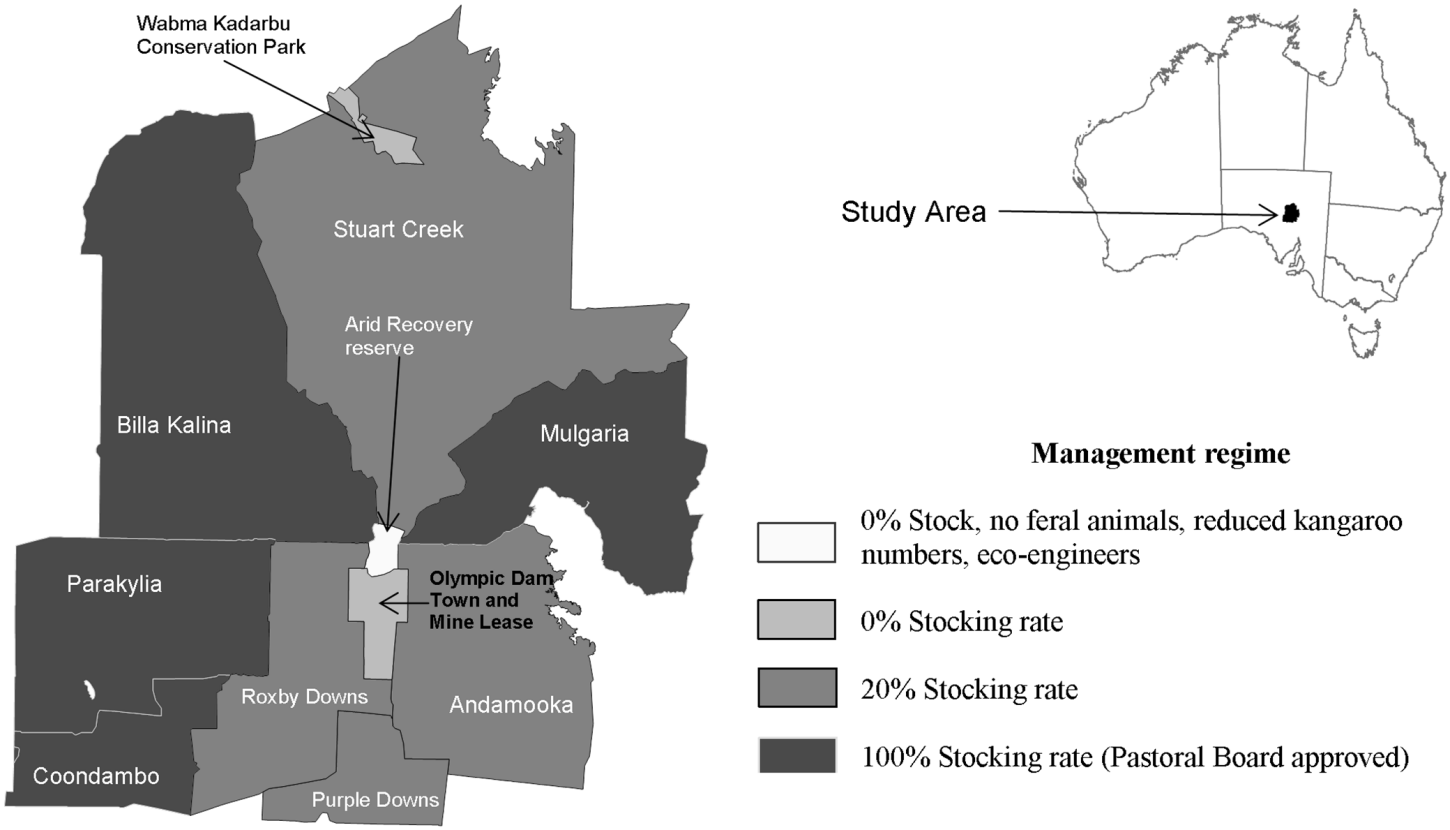
Location, land tenure and management change regimes within the study area.

The study area included all or parts of 15 leases held under different forms of land tenure (Fig 2), with each lease managed differently. They included nine commercially run Pastoral Leases, two different mining leases, a Country Township, a Conservation Park, an ungrazed railway siding and the Arid Recovery Reserve (Fig 2). The 15 leases are divided by fences of various types into 190 management units or paddocks, each sharing boundaries with at least one other paddock. The study area is centred on the BHP-Billiton Olympic Dam mine/Roxby Downs Town Lease and the Arid Recovery Reserve.

Most of this region has been grazed by introduced stock for over a century, originally by sheep, although cattle have become more predominant, especially on Pastoral Leases south of the Dog Fence (where wild Dingos are exterminated to protect stock) since 1980 [29]. Since the mid-1980s, with the introduction of the Olympic Dam mining lease, grazing management began to change, from predominantly full grazing regimes at the stocking limits set by the Pastoral Board of South Australia for every Pastoral Lease. Many paddocks of this study area are now managed sustainably with light grazing pressures, while others are still grazed according to stocking limits set by the Pastoral Board.

The Olympic Dam mine lease, Roxby Downs town lease, Wabma Kadarbu Conservation Park and parts of the Arid Recovery Reserve have been ungrazed by domestic stock since 1986, though rabbits and kangaroos are still present [29]. In 1995 Western Mining Company (later acquired by BHP-Billiton) acquired four Pastoral Leases in the region (Fig 2): Roxby Downs, Andamooka, Purple Downs and Stuart Creek Stations. Stock were removed entirely from one quarter of the paddocks within the leases that had been heavily grazed prior to acquisition, including the largest paddocks of Roxby Downs and Andamooka Stations. The remaining paddocks within those leases have since been limited to no more than 20% of the stocking rate set by the Pastoral Board of South Australia [30]. Historically the southernmost paddock of Stuart Creek has received little grazing pressure due to a lack of reliable waterpoints for stock, while the northern-most paddocks were heavily stocked with sheep by early pastoralists in the late 19^th^ century; these areas subsequently lost significant perennial vegetation and top soil around the plentiful water of the mound springs. The Andamooka mine lease is free from domestic stock, though it is intensively mined by numerous lease holders for opal, which causes considerable disturbance to soil and vegetation.

The Arid Recovery Reserve has had all grazing pressure from feral animals and domestic stock removed. This was done in several stages, starting in 1997 when Rabbit Haemorrhogic Disease reached the area, reducing rabbit numbers from ~50–300 rabbits/km^2^ to ~1–2 rabbits/ km^2^ [31], leading to a significant increase in seedling survivorship of perennial plant species during our study period [29]. The main exclosure of Arid Recovery was the first to be fenced, with domestic stock and rabbits removed by 1998 [29]. In the first, second and third expansions of Arid Recovery, rabbit grazing pressure was gradually removed by 2001 [32]. The fourth expansion rabbit removal program began in 2003 and took longer due to logistical constraints, with rabbit numbers deemed to be insignificant at 10 individuals or less in 26 km^2^ between September 2006 and May 2008 [32]. Several species of endangered native animal that are otherwise extinct in this study region were reintroduced to Arid Recovery between 1998 and 2001, including the Burrowing Bettong (*Bettongia lesueur*), Greater Bilby (*Macrotis lagotis*), the Greater Stick-Nest Rat (*Leporillus conditor*) and the Western Barred Bandicoot (*Peremeles bougainville*) [29]. These Great Bilby and Burrowing Bettong have since been described as “ecosystem engineers” as their burrowing activities positively increase soil quality and hence vegetation recovery [33], thus aiding in the restoration of the Arid Recovery paddocks during our study period.

The other Pastoral Leases are all privately held. Little is documented about the management practices of these leases. It is assumed for this study that the management of each does not exceed the upper stocking limits set by the Pastoral Board of South Australia. It is noted that Billa Kalina Station is a family owned lease, with a goal to preserve the country for the future by closely monitoring stock numbers and seasonal conditions (http://www.outbacklakessa.com.au/billakalina.htm accessed 3rd November 2014).

Amongst the 15 leases we identify four main changes that have occurred in management regime from the original region-wide full stocking, beginning in the mid-1980s: Ecosystem Restoration (ER); Complete Stocking Removal (CSR); Major Stocking Reduction (MSR); Full Stocking Maintained (FSM) (Fig 2).

### Remotely-sensed data

Two satellite image dates were chosen for analysis, based on rainfall records from the Bureau of Meteorology (BOM) for four pastoral stations throughout the study area. We chose imagery from November 2002 and October 2008 for change comparison, both of which followed periods of relatively low rainfall with preceding good summer rainfall events (Fig 1). In our analysis this combination is desirable for two reasons; summer rainfall events are more prevalent in this region and usually lead to considerable growth of native grasses, allowing maximum recovery of long term biomass from grazing pressure, while the dry period means that short term green-up of weed species following rainfall, is absent, and doesn’t adversely affect cover measurements in our imagery.

**Fig 3:**
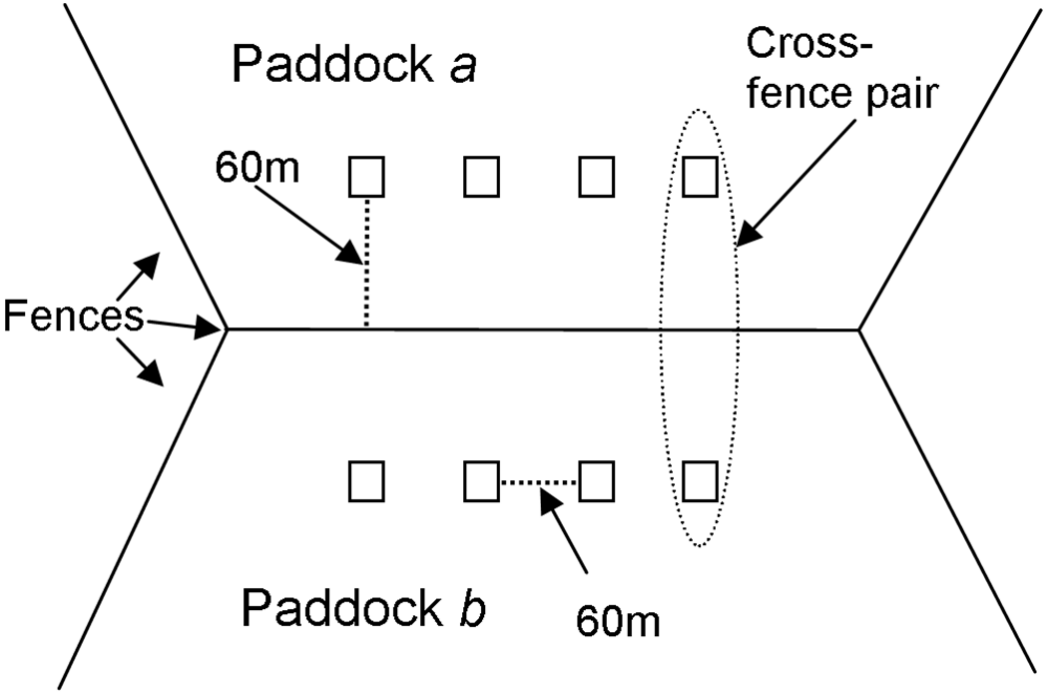
Layout of the GIS sampling regime for cross-fence comparisons.

Four adjoining Landsat Enhanced Thematic Mapper (ETM) and Thematic Mapper (TM) tiles were acquired for each date from the USGS GLOVIS website. The tiles were from the square of Rows 99 and 100 and Paths 80 and 81. In order to minimise cloud cover, dates for each set of four images were chosen from within the same month, though not necessarily from the same acquisition date. Landsat ETM imagery was used for November 2002, while TM was used for October 2008, due to the SLC error in later ETM imagery. No rainfall fell between acquisition dates for the set of four images. A correction to top of atmosphere reflectance [34] was applied. No additional atmospheric or radiometric corrections, such as BRDF corrections, need to be performed, as the nature of the cross-fence analysis means that image pixel pairs are subjected to the same atmospheric and sensor conditions, and if multiplicative, mathematically cancel out in the calculation of cross fence ratios (*r* in Eq 1 below). The fact that such image corrections are not an required for this methodology is indeed one of its great strengths. Imagery can be used in relatively raw forms, and can even include radiometrically or temporally un-matched mosaics, as long as image joining is not conducted along fence lines causing bias in the calculation of *r*. Further geometric corrections were not performed; instead we allowed for possible imagery misalignment in our sample design (Fig 3). Mosaicking of image tiles was performed with selection of overlapping areas preferring tiles with lower levels of cloud cover.

Many vegetation indices are now used for determining vegetation cover and soil exposure from satellite image data. Commonly, vegetation indices such as the Normalised Difference Vegetation Index (NDVI) [35] are used in arid areas to derive information about vegetation abundance, growth and distribution. Experience has shown that this index, while excellent for describing green-up of vegetation after rainfall events, is inadequate at describing perennial vegetation levels, particularly in southern and central arid Australia [6], [36] due to the low levels of green photosynthetic plant material present, the dominance of woody species and chenopod shrubs, and the short green-up periods of perennial grasses prior a long period in a dried but functional state. Various other indices for Landsat imagery have been suggested, such as PD54 [6] and the Soil Adjusted Vegetation Index (SAVI) [37], to help deal with lack of green photosynthetic vegetation, and also bias that arises due to varying background soil colour across an image. However these indices require subjective interpretations of soil lines or correction factors [6], [37]. Spectral unmixing techniques [7], [9], [10] can prove useful for determining vegetation cover levels in the arid zone, although the 250m ground sample distance of MODIS imagery is too large to be useful in smaller paddocks in the cross-fence comparison methodology.

In order to minimise these limitations associated with vegetation indices, we chose to use the red band of the Landsat TM imagery as an index of vegetation cover and soil exposure. Due to the bright red/brown nature of exposed soils in this region and the general absorption of red wavelengths by both green and dry plant biomass, the inverse of red band can be used to describe relative vegetation cover levels [38], [39], [40].

### Fenceline data

Fenceline data used was a combination of polygon, polyline and coverage GIS files provided by the South Australian Government (Department of Environment and Natural Resources, Pastoral Management Branch) and Arid Recovery Ltd. These layers were aggregated into a shapefile in GIS and updated to include fence positions for 2008. Fencelines that were no longer functioning or had been removed in the last decade were included, as historic management within these boundaries affects present day conditions. It is noted when aligning fence boundaries from different sources that there were several minor discrepancies of up to 50 metres between data sets; in these cases the mean position of the boundary from the two sources was chosen for the fenceline position. Each of the 190 paddocks was given a unique identifier.

### Cross-fence analysis methodology

The cross-fence comparison methodology, as outlined in Kilpatrick et al [16] was used for analysis. This method uses a measure or index of vegetation condition *c*_*a*_ and *c*_*b*_, at both sides of fences for paddocks *a* and *b*, respectively. Cross-fence sample pairs of single pixels were extracted in the GIS, with sample points located in the paddocks 60m from the fenceline, at sample intervals of 60m along the fence (Fig 3) using the “sampleperppointsalonglines” tool in Geospatial Modelling Environment (version 0.5.2 Beta, Hawthorne L Beyer, 2010). The 60m by 60m sample distance was chosen in order to account for fenceline mapping errors, georectification errors in Landsat imagery, to avoid sampling pixels more than once and to avoid sampling roads and tracks that usually border fencelines in this region. A distance between samples in a pair of 120m was considered adequate for controlling localized variables such as soil, landcover type, vegetation type and rainfall spatial variability in this region. This sample spacing was based on visual inspection of landscape features in true and false colour composites of the Landsat imagery and semivariance analysis of an ALOS PALSAR 2.5m panchromatic image of a significant central portion of the study area. The resultant semivariograms indicated that directional sill values are not reached until at least 400m, which is indicative of the minimum spacing of the approximately east-west sand dunes that occur in parts of the region. Consequently the 120m between samples in a pair is adequate control for general natural spatial variation.

Fenceline intersections between two or more paddocks were avoided in the sampling regime, as were sections of fenceline with sharp corners, in order to avoid sampling the same paddock twice. Only boundaries internal to the study area were sampled in the GIS, with external boundaries ignored. In our study region this had the advantage of excluding paddock boundaries with the main geographic anomalies such as salt lakes, which otherwise would have needed to be excluded from the analysis.

The *relative condition ratio* between paddocks *a* and *b* (Fig 3), *r*_*ab*_, along the fenceline section that is shared by paddocks *a* and *b*, is characterised as geometric mean of condition ratios between *c*_*ak*_ and *c*_*bk*_, for each of *l* cross-fence sample pairs as

$${\text{r}}_{\text{ab}}={\left(\prod_{\text{k}=1}^{\text{l}}\frac{{\text{c}}_{{\text{a}}_{k}}}{{\text{c}}_{{\text{b}}_{k}}}\right)}^{\frac{1}{\text{l}}}$$ (1)

The *paddock performance p*_*i*_ for any paddock *i* can be estimated relative to all other paddocks as the weighed mean of all ratios as:

$${\text{p}}_{i}=\frac{1}{{\text{n}}_{i}}\sum_{\text{j}=1}^{\text{t}}{\text{r}}_{\text{ij}}{\text{p}}_{j}$$ (2)

where

*r*_*ij*_ = 0 when *i* = *j* or if the *i* ^*th*^ and *j* ^*th*^ paddocks are not neighbours

*n*_*i*_ = number of neighbours of the *i* ^*th*^ paddock

*t* = total number of paddocks in the system

The equations for *p*_*i*_ (Eq 2) constitute a system of *t* linear equations with *t* unknowns that can be solved. We used Python scripting to generate the matrix of the *r*_*ij*_ estimates and SVD (Singular Value Decomposition) in MATLAB to solve the equations. SVD is numerically stable in solving large systems of homogeneous linear equations, with high numbers of variables and zero values [41], which is important as most *r*_*ij*_ and hence *p*_*i*_ values in our system of homogenous linear equations are zero; as most paddocks only share fencelines with a few other paddocks in the system.

### Paddock statistics

We converted the 190 raw paddock performance scores, *p*_*i*_ of solutions to the homogenous linear equations for each date, to a rank of paddocks, in order to make results more meaningful and easier to interpret for comparison with recent management changes in the region, which themselves are not quantifiable. This also makes comparisons of paddock rank between the two dates possible. Changes in rank were calculated for each paddock between the two imagery dates and mapped. We performed a Welch’s two-tailed t test of rank change between the FSM and MSR management regimes as these were the two major land use changes in the region.

## Results

### Landcover

**Fig 4:**
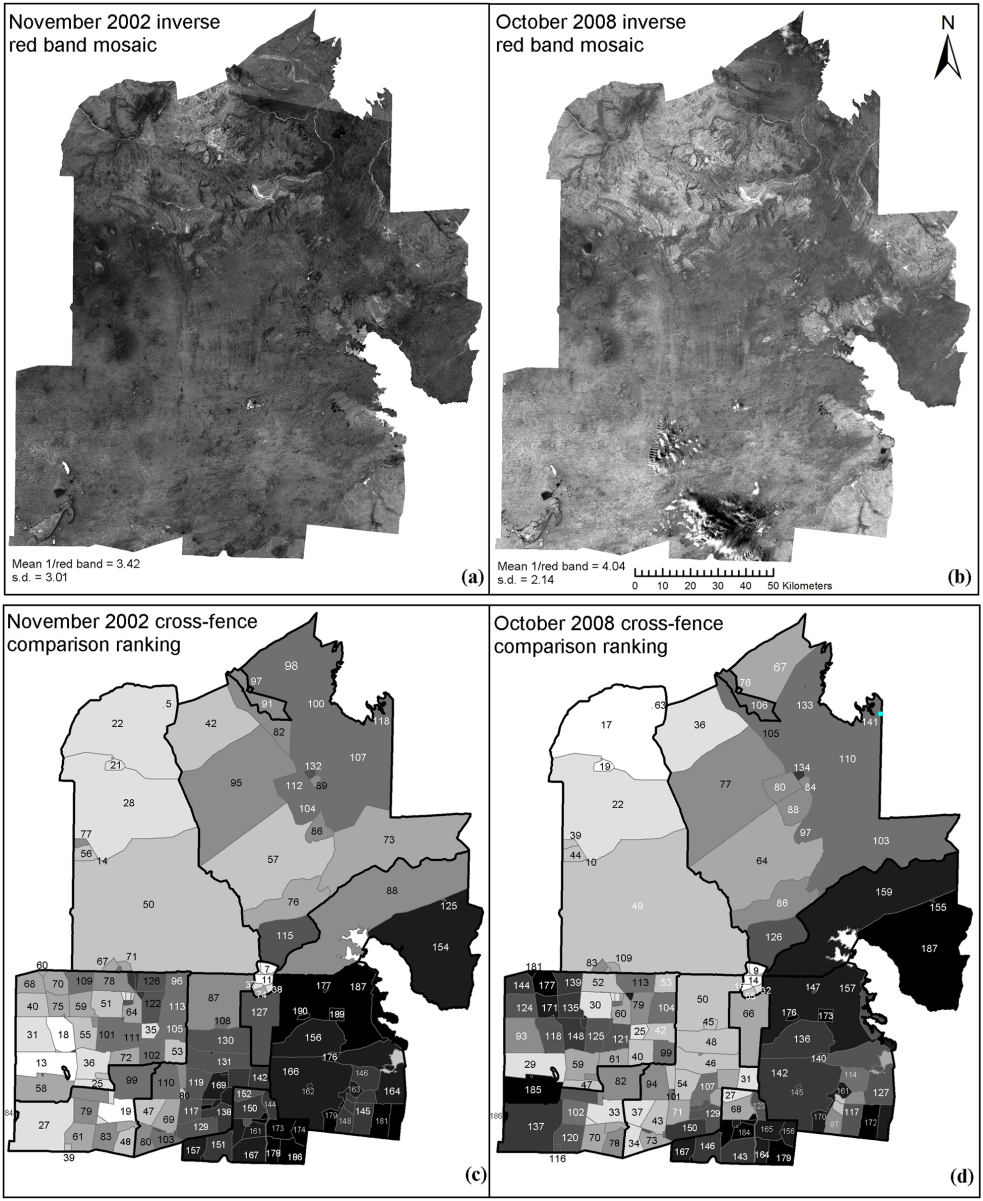
(a) November 2002 inverse red band mosaic. (b) October 2008 inverse red band mosaic. (c) November 2002 cross-fence comparison ranking. (d) October 2008 cross-fence comparison ranking. For (a) and (b), a 1 standard deviation contrast stretch was used in display. Dark pixels represent low levels of vegetation index and, hence low levels of vegetation cover. Bright pixels represent high vegetation index and cover. For (c) and (d), dark paddocks represent low rankings, hence poorly performing paddocks, while bright paddocks indicate high rankings and highly performing paddocks in the system.

Fig 4a and 4b show the vegetation cover of the study area in November 2002 and October 2008 respectively, as indicated by the Landsat inverse red band index. Vegetation cover varies widely in magnitude and spatial distribution throughout the study area, with the same landscape patterns evident on both image dates, such as the stony tableland country in the northern third, high cover in creek lines and what appears to be low cover surrounding cane grass swamps in the western portion. Other features such as sand dune/swale repetition can be discerned in both epochs of imagery. Such spatially variable vegetation cover associated with landsystems means that using raw vegetation indices, or averages of indices for each paddock, would be a misleading guide to land condition, and certainly wouldn’t allow meaningful comparisons to be made between paddocks.

### Cross-fence comparison rankings and the effects of management

**Fig 5:**
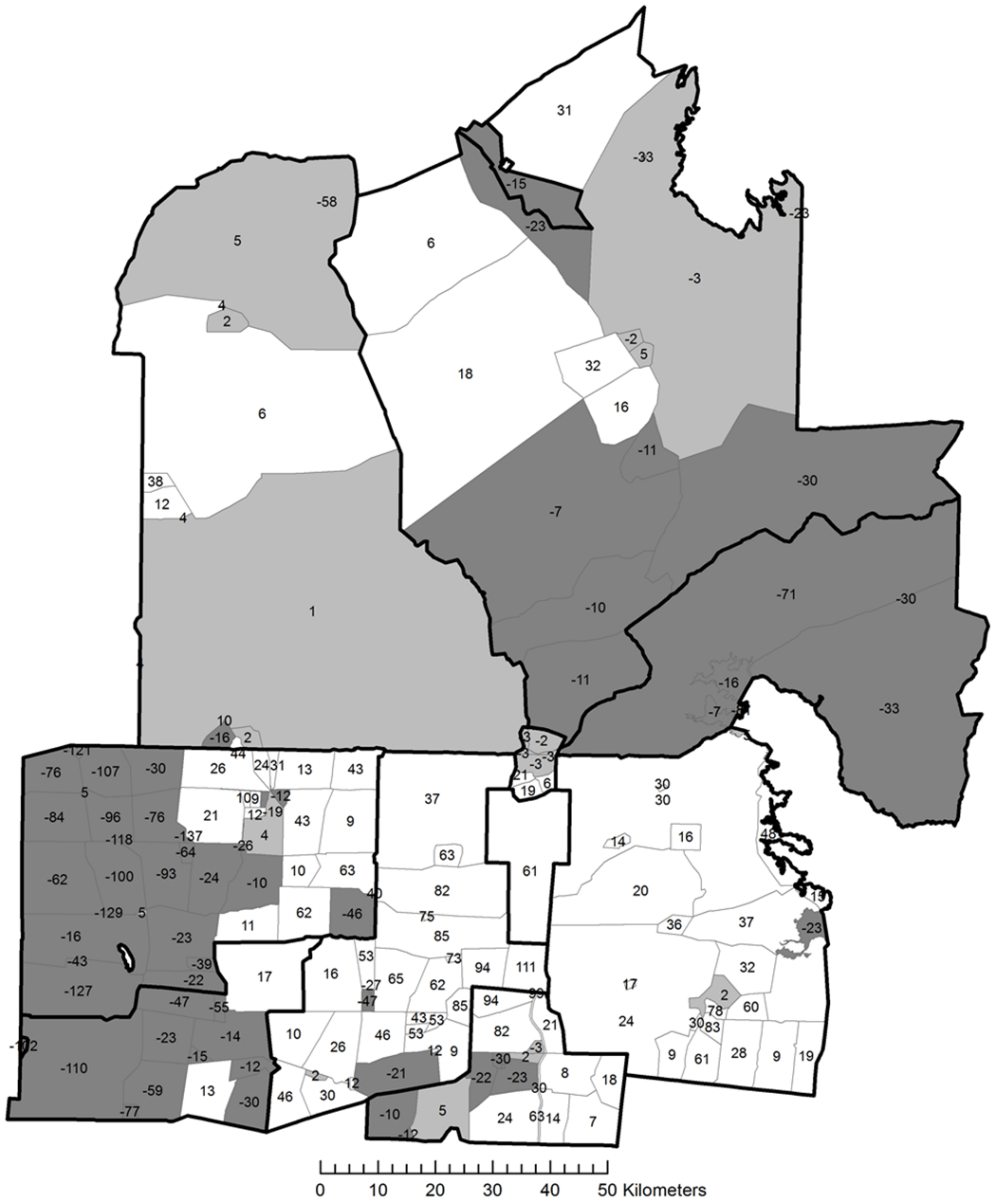
Spatial representation of change in paddock rankings between November 2002 and October 2008. Positive numbers are paddocks with increased ranking, whilst negative numbers are paddocks with a reduced ranking. Changes of +/- 5 in rank between the two dates are considered to be minor and not significant. Shadings denote positive (light), no significant change, and negative changes (dark).

Fig 4c and 4d show the cross-fence comparison ranking for the two image dates. Most paddocks change in rank between these two dates. Fig 5 displays the change in rank between 2002 and 2008, showing the spatial distribution of change with relation to station boundaries and this can be compared spatially to the management regimes shown in Fig 2. The rank change is not uniform within station boundaries, with some stations showing more heterogeneity than others. Overall the majority of stations show a general uniformity in their rank behaviour. General increases in rank occur in the pastoral stations of Andamooka (MSR), Roxby Downs (MSR) and Purple Downs (MSR), as well as the Olympic Dam Town (CSR) and Mine Leases (CSR). The Arid Recovery Reserve (ER) also shows an overall positive increase in ranking, with three paddocks showing an increase, while the remainder show no significant difference. Pastoral stations with an overall neutral or minor change in rank are Billa Kalina (FSM) and Stuart Creek (MSR). Pastoral stations with an overall decrease in rank are Parakylia (FSM), Mulgaria (FSM), Coondambo (FSM), and also the Wabma Kadarbu Conservation Park (CSR).

The sample sizes of the CSR and ER management regimes are low, and given their relatively high rank positions in both dates, we excluded them in further statistical tests of rank change. However, there was a highly significant difference in rank changes between the MSR and FSM management regimes (P = 0.00000132, Welch’s T Test, Two-tailed).

### Expected and observed changes in paddock condition

**Fig 6:**
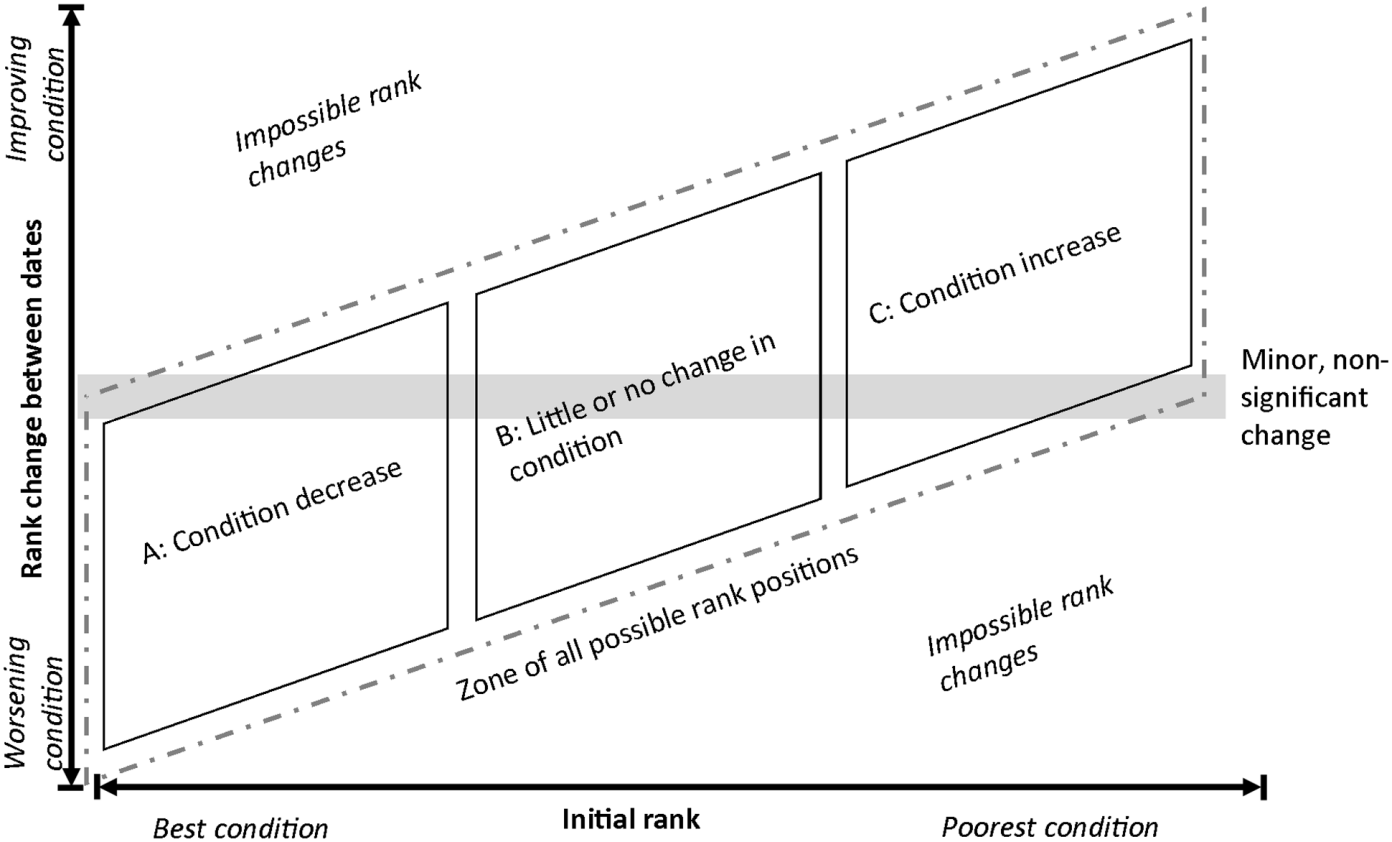
Schematic of expected and possible changes amongst the regimes.

Fig 6 presents the conceptual framework for interpretation of paddock ranks and rank changes between the two dates. Possible rank changes between the first and second image date (y-axis) are compared with ranking on the first image date (x-axis). A limited number of changes in paddock rank are possible. Paddocks with the best condition on the first date (Fig 6; category A) can either not change, or decrease in rank. Paddocks with intermediate ranks can either decrease or increase in rank (Fig 6; category B), while the paddocks with the lowest ranks (poorest condition) on the first date can only stay the same or improve by the second date (Fig 6; category C). With four different management regimes in place between the two dates, we predict that those with the better management will either stay the same or increase in rank relative to those with poor management.

**Fig 7:**
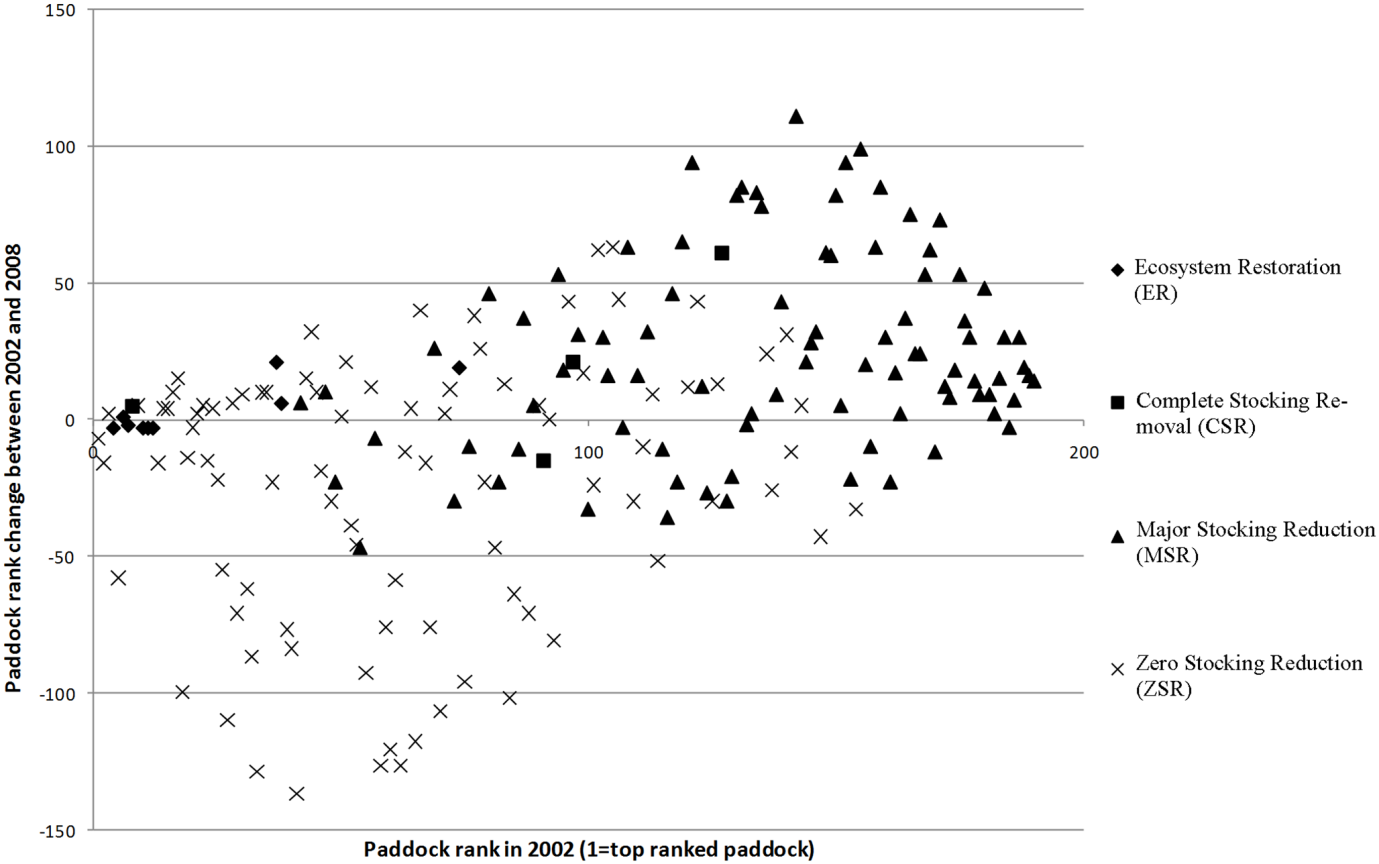
Graphical representation of change in rank over time, in relation to the four management regimes, for the 160 paddocks in the study are, a against initial rank and management regime.

Fig 7 displays the results of the cross-fence analysis. The relationship of original paddock rank of paddock condition index, *p*_*i*_, and change in ranks of *p*_*i*_ during the study period, is shown for all paddocks in the four management regimes during the period. The majority of paddocks under the FSM management regime show either a decrease in rank or a relatively insignificant (neutral) rank change of +/- five (5) rank positions. Paddocks with the MSR regime mostly show positive improvements. Paddocks under the two CSR regimes showed either a neutral or positive response. A neutral response does not necessarily have negative connotations. If a paddock was already one of the highest ranked on the first image date, it can have only two possible outcomes: no significant change, or decrease in rank (Fig 6; category A). Thus no significant change is a good outcome; the paddocks are still performing better than all others within this study area. A paddock that is amongst the lowest ranked paddocks in the first image date also only has two options; to increase, which is the positive outcome, or no significant change, which is undesirable (Fig 6; category B). Paddocks towards the middle of the rankings have increased or decreased in rank. Consequently the results shown in Fig 7 generally confirm the expectations of how the different paddocks would perform under different management regimes.

#### Ecosystem Restoration (ER)

The Arid Recovery Reserve paddocks (ER) were amongst the highest ranking paddocks on both image dates (Fig 4), suggesting that since the Reserve was established in 1997, significant positive improvement in vegetation cover likely took place during the five years prior to the first image date, although further improvement between 2000 and 2008 was shown in the southern three paddocks. The northern paddocks of Arid Recovery show no significant change: being among the highest ranked paddocks in the study, they could only either remain the same, or decrease in rank relative to the others (Fig 7). Both of these results are as expected, given the total grazing reductions, complete control of feral grazing pressure from introduced rabbits and ecosystem improvements in the Arid Recovery Reserve as previously noted in the literature.

#### Complete Stocking Removal (CSR)

There were two main paddocks under the CSR regime on both image dates. One is the Olympic Dam Town and Mine Lease, which was originally destocked in 1986, though is still grazed by rabbits and often heavy kangaroo numbers. Given the difference in management between this paddock and the Arid Recovery reserve, it is not surprising that it has a lower ranking in both 2002 and 2008 than Arid Recovery. It is also notable that it still shows an improvement in ranking between these two image dates, despite having been destocked of sheep and cattle 16 years prior to 2002; this reflects both the lag time of response to de-stocking in arid zones, but also the significant effects that rabbit and kangaroo numbers have on vegetation cover and recruitment. For example, whilst Mulga trees (*Acacia aneura*) have regenerated inside the Arid Recovery Reserve since rabbits and stock were removed, little recruitment has occurred in the adjacent mine lease [42].

The second paddock within the study area with no stock is Wabma Kadarbu Conservation Park, which unlike the Olympic Dam Mine and Town Lease, shows a significant decrease in rank (Fig 5, -15 ranks) between the two image dates. The most likely explanation is that this paddock was one of the earliest areas grazed in the region when pastoralists first arrived in the late 19^th^ century. During this period, waterpoints were yet to be developed, and so grazing was extremely intense around mound springs, the only constant natural source of water in the region, resulting in catastrophic loss of vegetation cover and soil. Despite being destocked, it is likely that this and other paddocks containing mound springs will take an extremely long time to recover to a more vegetated state due to loss of soil and seedbank, or may never do so, having transitioned to a new ecological state. In terms of cross-fence rankings and our results, it is quite possible that due to a poor baseline and ability to recover, even though it may have recovered to some small extent between our two image dates, it actually worsened in condition relative to many of the other paddocks within our closed system.

#### Major Stocking Reduction (MSR)

The southern three stations under the MSR regime; Roxby Downs; Purple Downs; and Andamooka, all showed a clear increase in rank between the two image dates, as predicted after the reduction in total grazing pressure. Stuart Creek, the other station that was MSR over the same period, however, has a mix of paddocks that increased, decreased, and did not change. Reasons for some of these paddocks not increasing are unclear, though it is possible that, like the Wabma Kadarbu Conservation Park, they were overgrazed at some stage during their history, leaving them with a lower ability to recover following a reduction in grazing pressure.

#### Full Stocking Maintained (FSM)

The majority of the paddocks in the stations Coondambo, Parakylia and Mulgaria under the FSM regime show a decrease in rank between the two image dates (Fig 5). Some paddocks in Coondambo and Parakylia show no change or some improvement, particularly in the eastern section of Parakylia. The cause of this heterogeneity of rank change is unknown; however, possible resting of paddocks and renewed grazing of others within each station is likely to have been undertaken by the managers, spreading their grazing quotas around based on condition before November 2002.

Billa Kalina station shows either no change or increased rank in its constituent paddocks (Fig 5), despite its FSM regime, though this result does conform to the conservative management statements expressed by the station managers on their website. Another possible reason for the high ranking could be that paddocks within this property are particularly large, resulting in more areas remote from grazing existing in proximity to the paddock boundaries, driving its ranking upwards.

## Discussion

### Interpreting and applying paddock rankings

There are several factors that need to be considered in interpreting changes in paddock rankings. Firstly, with this analysis all paddocks within the system were ranked relative to one another, with no known baseline or reference paddock; we are unable to identify a paddock within the area with documented evidence of unchanged condition over the study period. This results in a conundrum; without a benchmark paddock of known, zero (or near) change in condition for comparison, we can’t determine whether paddocks have decreased or increased in actual condition. Thus, it is possible, for example, that the best paddocks, such as those within Arid Recovery (ER), have decreased in vegetation cover, yet they will still appear excellent in the analyses. Or, conversely, the lowest ranked paddocks and those that have decreased in ranking might have improved in real condition over this time period, although our analysis will show them to have decreased in rank.

The key is the relative nature of the ranking system, and in future operational use it would be desirable to use benchmark paddocks to assist in interpreting the results. After conducting the remotely-sensed cross-fence analysis, the ranking of these reference or benchmark can be identified, and all others ranked relative to them.

### The closed loop problem

With known benchmarks unavailable, it is difficult to avoid a situation where some paddocks increase in actual condition on the ground, yet show a decrease in rank in the analysis, or vice versa. The reason for this is that in a system of *t* paddocks, the maximum possible rank places that a paddock can change is *t*-1, depending on its original rank; and that the sum of all the changes in rank must be equal to zero. This results in a situation where paddocks that have improved in real condition, may not have improved in the paddock ranking, as they did not improve sufficiently, relative to their peers. In this situation, time series of rank changes over time, derived from multiple dates of imagery, with known benchmark paddocks included in the analysis, would aid in interpretation of paddock performance.

### The piosphere effect, wind direction, and grazing distribution

While this methodology by design controls for local environmental variables, it does not control for factors that influence the distribution of grazing animals within a paddock, which influences cover levels at each sample point. Intensity of grazing pressure is known to decrease radially outwards from waterpoints [27], and domestic stock such as sheep are known to graze preferentially into the wind [28]. These variables could be implemented as weighting factors [16], if suitable models of grazing distribution were available [43], [44], and if preferential grazing into the wind were quantified. However the latter has not yet been documented.

Despite lacking a weighting model for the piosphere effect, the cross-fence methodology shows the effects of changes in management, with Fig 6b confirming predicted changes, especially given the relatively limited knowledge available on the management within the paddocks in question. Accounting for grazing distribution of domestic stock would affect our results. As the Arid Recovery Reserve (ER) and several other paddocks in the region, including the Olympic Dam Town (CSR) and Mine Lease (CSR) and Wabma Kadarbu Conservation Park (CSR), do not contain domestic stock and thus do not contain active grazing gradients, their derived values and rankings would be driven in a positive direction. Paddocks that are particularly large and/or contain a higher density of water points, would likely rank lower if such weighting models were implemented.

### Timing of imagery and changes in fenceline layouts, management regimes and lag-time

Differences in fenceline layout between November 2002 and October 2008 may have affected the results of this study, particularly for the first image date. While the majority of fences were unchanged between these two dates, the northernmost paddock of Arid Recovery (Fig 4c, rank 7) (ER), was fenced in February 2005; thus it did not exist in our first image. Analysing rank changes for this paddock in the context of management holds little meaning, although it is interesting that this paddock effectively remained stable in ranking, possibly due to a legacy of good grazing management in the corners of the original paddocks from which it was fenced. The exact effects of this “ghost paddock” on other paddocks are uncertain, although they are likely small. The minimisation of spatial variation inherent in the cross-fence design means that if a non-existent fenceline is included in the analysis, the vegetation index values either side of the “fence” will be approximately equal and result in a mean cross-fence ratio (*r*_*ab*_ *)* of one, thus imparting no net effect on the equations for the paddocks in question. Effectively, the “ghost paddock” could be removed from the calculated paddock rankings, and the rankings adjusted accordingly, with no ill effects.

### Field validation of the cross-fence comparison methodology

It is exceedingly difficult to isolate the effects of management on rangeland condition. Determining these effects with field studies requires an extensive, spatially comprehensive sampling regime encompassing a number of paddocks, randomly chosen throughout a study system. The field observations must match in sampling extent and component what the remote sensing data “sees” [45]. Currently such intensive field data is unavailable. The required data would not be restricted to vegetation cover levels, but also include a wealth of information regarding vegetation community composition and condition, soil degradation, and ecosystem structure and status would be required to make such assessments. A range of indices such as a biodiversity intactness index [46] could be compared to this cross-fence comparison methodology in order to assess whether it is sufficiently robust to be used as a stand-alone monitoring method for rangelands.

## Conclusions

The analysis presented shows an effective proof-of concept that the cross-fence methodology may provide new information for rangeland management. It informs managers about the consequences of rangeland management that is decoupled from natural spatial and temporal variability, at an information scale appropriate for decision making. Thus it provides a potentially useful addition to condition indicators to inform long-term strategies of stock management. Conceptually, the method is based on cross-fence comparisons, an approach long used by rangeland managers. However our methodology allows comparison of a paddock with all others in a region, not just its immediate neighbours, providing a much more comprehensive overview of land management outcomes. The method could be applied to single properties, allowing managers to assess relative condition of paddocks, or at regional scale to assess broad management regimes, as has been demonstrated in this paper. An additional strength of the method is that it can be applied to single dates of remotely-sensed imagery to assess relative condition at particular times, or to multiple dates to assess changes in condition. Unlike other recently published methods for assessing rangeland condition with remote sensing, our approach does not require long time-series of calibrated images, and can be applied to many types of earth-observation imagery, without extensive pre-processing, due to its ability to spatially control for such influences as BRDF and background soil colour.
